# Supplementary material for: Causal interplay between lactose intolerance and gut microbiota: a combined bidirectional Mendelian randomization and in vivo validation study
Source: Front Nutr. 2026 Jun 1;13:1803337. doi: 10.3389/fnut.2026.1803337 (PMC13265576; doi:10.3389/fnut.2026.1803337)
Supplement: Supplementary file 1 [file Data_Sheet_1.zip › supplementary materials/Forward/leave one out cross validation/ebi-a-GCST90027512.finngen_R12_E4_LACTONAS.pdf]

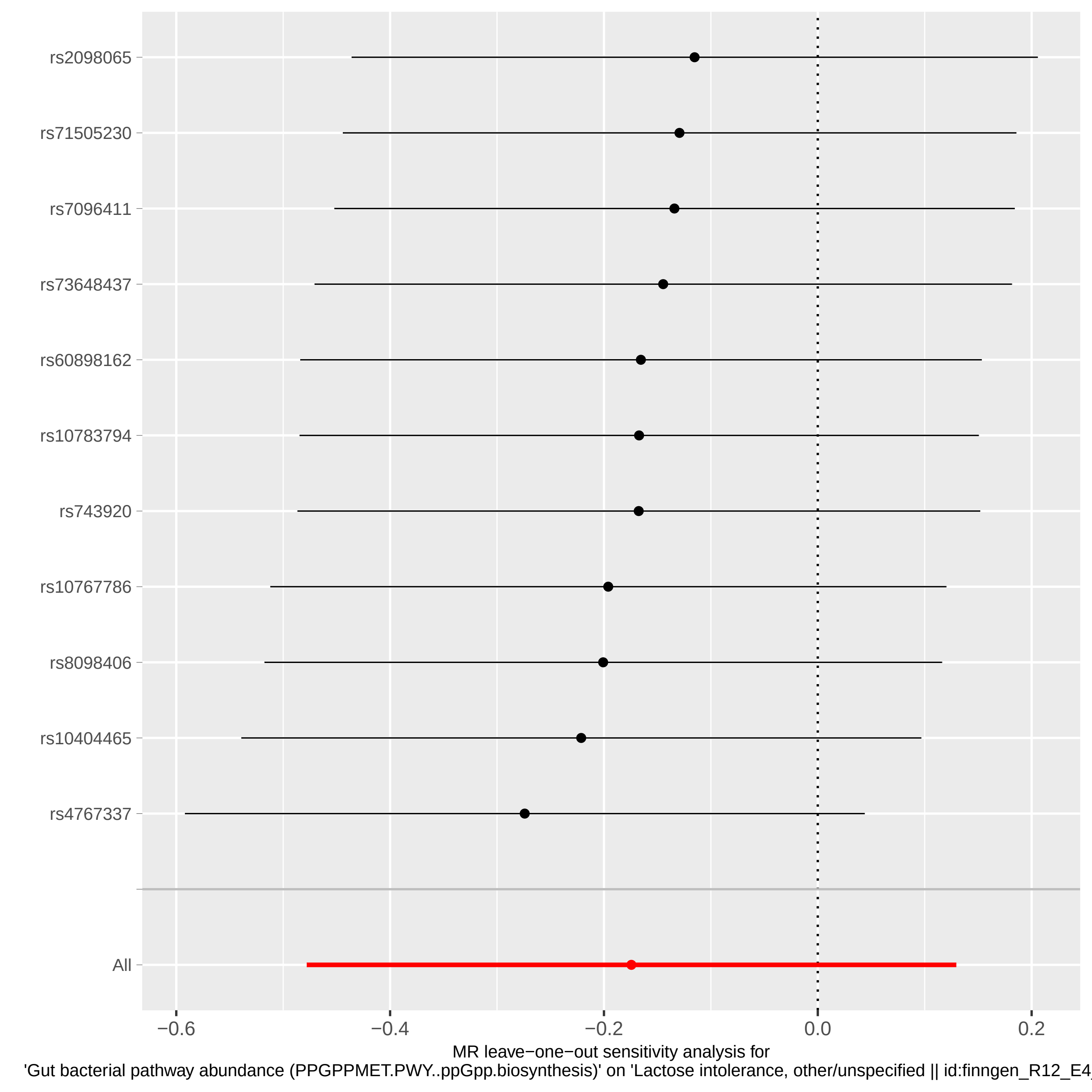
rs2098065
rs71505230
rs7096411
rs73648437
rs60898162
rs10783794
rs743920
rs10767786
rs8098406
rs10404465
rs4767337
All
−0.6
−0.4
−0.2
0.0
0.2
MR leave−one−out sensitivity analysis for
'Gut bacterial pathway abundance (PPGPPMET.PWY..ppGpp.biosynthesis)' on 'Lactose intolerance, other/unspecified || id:finngen_R12_E4
